# Sensor-Based Frailty Assessment Using Fitbit

**DOI:** 10.3390/s24237827

**Published:** 2024-12-07

**Authors:** Mohammad Hosseinalizadeh, Mehran Asghari, Nima Toosizadeh

**Affiliations:** 1 Department of Biomedical Engineering, School of Graduate Studies, Rutgers University, Newark, NJ 07107, USA; 2 Department of Rehabilitation and Movementformul Sciences, School of Health Professions, Rutgers University, Newark, NJ 07107, USA; 3 Department of Neurology, Rutgers Health, Rutgers University, New Brunswick, NJ 07103, USA; 4 Brain Health Institute, Rutgers University, New Brunswick, NJ 07103, USA

**Keywords:** frailty assessment, wearable sensor, Fitbit smartwatch, motor performance, heart rate (HR) monitoring

## Abstract

This study evaluated the reliability of Fitbit in assessing frailty based on motor and heart rate (HR) parameters through a validated upper extremity function (UEF) test, which involves 20 s of rapid elbow flexion. For motor performance, participants completed six trials of full elbow flexion using their right arm, with and without weight. Fitbit and a commercial motion sensor were worn on the right arm. For HR measurements, an ECG system was placed on the left chest alongside the Fitbit on the left wrist. Motor parameters assessing speed, flexibility, weakness, exhaustion, and HR before, during, and after UEF were measured. A total of 42 participants (age = 22 ± 3) were recruited. For motor parameters, excellent agreement was observed between the wearable sensor and Fitbit, except for flexibility (ICC = 0.87 ± 0.09). For HR parameters, ICC values showed weak agreement between ECG and Fitbit for HR increase and recovery (ICC = 0.24 ± 0.11), while moderate to stronger agreement was seen for mean HR during baseline, task, and post-task (ICC = 0.81 ± 0.13). Fitbit is a reliable tool for assessing frailty through motor parameters and provides reasonably accurate HR estimates during baseline, task, and recovery periods. However, Fitbit’s ability to track rapid HR changes during activity is limited.

## 1. Introduction

Frailty is a clinical syndrome prevalent among older adults, characterized by diminished strength, endurance, and physiological function, which increases an individual’s vulnerability to stressors. Frailty is associated with a higher risk of adverse health outcomes, including falls, hospitalization, disability, and mortality [1,2,3,4], and it affects approximately 11% of community-dwelling older adults and as many as 30–70% of older surgical patients [5,6]. Identifying and managing frailty is crucial in clinical practice to improve patient outcomes. Despite its significance, frailty often remains underdiagnosed due to the lack of standardized and accessible assessment tools.

Motor function and heart rate variability (HRV) metrics play significant roles in assessing frailty. Motor performance, such as gait speed and grip strength, provide valuable insights into an individual’s physical function, muscle strength, and overall mobility, which are critical indicators of frailty [7]. Meanwhile, HRV is a key indicator of autonomic nervous system function and overall cardiovascular health, reflecting the balance between sympathetic and parasympathetic activity and the body’s ability to adapt to stressors [8]. Frailty is often linked to an impaired ANS and reduced cardiac function, both contributing to diminished physiological reserves [8]. Research has shown that frail and pre-frail individuals exhibit a smaller heart rate (HR) increase during physical activity and slower recovery times post-exercise; therefore, assessing HR dynamics alongside motor function offers a more comprehensive evaluation of frailty [4]. This combined cardiac and motor assessment approach has demonstrated improved sensitivity and specificity in frailty detection [4].

Previous research on frailty assessment has encountered several limitations, such as reliance on subjective measures and the need for specialized equipment and trained personnel. These constraints emphasize the need for more accessible and objective tools. Wearable technology, like the Fitbit smartwatch, offers a promising solution [9,10]. By continuously monitoring physiological and physical activity data, wearable devices can provide real-time and objective assessments of frailty. The increasing use of smartwatches in health monitoring has shown significant potential across various fields, including improving adherence to health-monitoring protocols in clinical studies [11] and enhancing disease management [12] and even stress management for veterans [13]. Additionally, smartwatches have been successfully integrated into rehabilitation programs for patients recovering from surgery [14,15] and managing cardiac conditions [16]. Their ability to provide real-time feedback, coupled with their accessibility, has allowed for personalized interventions that improve patient outcomes.

The aim of the current study was to evaluate the effectiveness of Fitbit, instead of commercial sensors, in frailty assessments through an upper extremity function (UEF) test while measuring motion and HR parameters. Motion and HR parameters measured using commercial sensors within the UEF test have been shown to be effective indicators of frailty status. The UEF test involves rapid elbow flexion, assessing motor performance such as slowness, weakness, inflexibility, and fatigue [17,18]. Previous validations have demonstrated the effectiveness of the UEF motor components in distinguishing frailty levels among older adults, using the Fried frailty index and the short-version Rockwood questionnaire as comparators [7,19,20]. Further HR measures, including HR changes during and after UEF, have demonstrated a strong association with the Fried frailty index [4]. The Fried frailty index is a well-established clinical tool that defines frailty based on five physical criteria: unintentional weight loss, self-reported exhaustion, low physical activity, slow gait speed, and weak grip strength [1]. A person is classified as frail if three or more of these criteria are present, pre-frail if one or two criteria are present, and non-frail if none are present. The short-version Rockwood questionnaire, meanwhile, is based on the concept of a frailty index, which measures the accumulation of health deficits (e.g., symptoms, signs, and laboratory abnormalities) across various domains [2]. It provides a more comprehensive assessment of frailty by considering cognitive, psychological, and social factors alongside physical indicators.

Building upon our previous research, the hypotheses for the current work were as follows: (1) motor performance parameters obtained from the UEF test using Fitbit will show an excellent intraclass correlation coefficient (ICC ≥ 0.8) with those measured by the commercial wearable sensor, and (2) HR measurements with Fitbit will exhibit an excellent ICC with those recorded by the wearable ECG device. Although we conducted this study among young, healthy participants, frailty assessment in the UEF task was based on differences in motion parameters. Using weights during the task, we aimed to generate varying motion parameters and HR measures that mimic the performance of frail individuals, allowing us to compare the ability of Fitbit and commercial sensors to capture both motion and HR data across a range of simulated frailty levels.

## 2. Materials and Methods

### 2.1. Participants

Participants were recruited from a local community, targeting young, healthy adults aged 18–30 years. Recruitment involved screening for general health status, and participants were excluded if they had (1) severe diagnosed motor function deficits (Parkinson’s disease, multiple sclerosis, or recent stroke); (2) severe upper-extremity disorders (i.e., elbow or shoulder fractures/tears, rheumatoid arthritis, crystal arthropathy, surgery, or any other upper-extremity disorder that could prohibit the UEF test); or (3) any other severe illness that, based on the judgment of the investigator team, prohibited the participant from performing the experimental tasks.

### 2.2. UEF Motor Test

**Figure 1:**
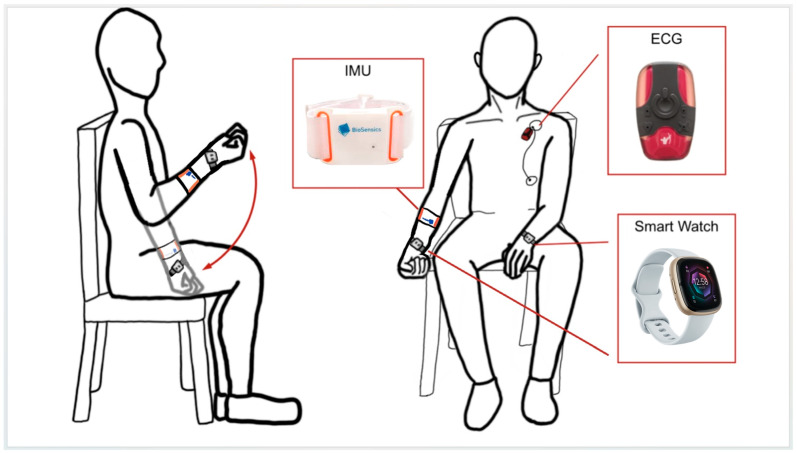
The UEF test setup schematic with IMU, ECG, and smartwatch sensors for motor and HR monitoring.

Details of the UEF validation and index development using commercialized sensors have been comprehensively explained in our previous work, and only crucial aspects of UEF regarding the measurement procedure are presented here [7,20]. For UEF, while sitting on a chair, participants performed six trials of full elbow flexion and extension as fast as possible for 20 s using their right arm (see Figure 1 for test setup schematic). Three of these trials were conducted with an additional weight to resemble different frailty stages. Before the test, the participants performed a short practice trial with their left arm to become familiar with the protocol. The protocol was explained to the participants, and they were encouraged only once, before elbow flexion, to perform the task as fast as possible. To ensure consistency, the same verbal instruction was used, and the participants were not further encouraged during the task.

A wearable sensor (triaxial gyroscope, BioSensics LLC, Cambridge, MA, USA, sampling frequency: 100 Hz) and a Fitbit^®^ Sense 2 (Fitbit Inc., San Francisco, CA, USA, gyroscope sensor sampling frequency: 100 Hz) were both worn on the dominant wrist to collect motion data. We recorded 20 s motion signals from both sensors, which were then filtered to remove noise and drift using a first-order high-pass Butterworth filter with a cutoff frequency of 2.5 Hz. Using these filtered signals, we calculated the frailty score based on our previous research and an algorithm we developed [20]. The frailty score ranges from 0 to 100, where 0 indicates no frailty, and 100 represents extreme frailty. The frailty score incorporates several parameters, such as speed, moment, flexibility, speed variability, and speed reduction (see Table 1 for parameter definitions). The frailty score and frailty sub-parameters were used to compare the motion signals of the wearable sensor and Fitbit.

### 2.3. HR Assessment

HR was continuously monitored during the UEF task using both a wearable electrocardiogram (ECG) system and a Fitbit. The wearable ECG system (360° eMotion Faros, Mega Electronics, Kuopio, Finland; ECG sampling frequency = 1000 Hz, accelerometer sampling frequency = 100 Hz) was used to collect HR data. Two electrodes were placed on the left chest, one on the upper mid-thorax and one on the inferior side of the left rib cage, to record a one-channel ECG, with synchronized accelerometer data ensuring the precise identification of the task start and end points. The Pan–Tompkins algorithm [21] was used for QRS peak detection for the ECG data, allowing the extraction of the RR intervals and the calculation of the HR outcomes. In addition to the ECG setup, the Fitbit was worn on the left wrist, while the UEF task was performed with the right arm to minimize motion artifacts. For both the ECG and Fitbit, HR was recorded throughout the task, as well as the baseline and recovery periods, which were defined as 60 s before and 60 s after the activity, respectively. For both the ECG and Fitbit systems, five HR-related parameters were extracted for analysis, including HR increase, HR decrease, mean baseline HR, mean task HR, and mean post-task HR (see Table 1 for parameter definitions).

### 2.4. Fitbit and Android App

We collected and processed the motion data from Fitbit by developing both Fitbit and Android applications. A prototype Fitbit app (https://community.fitbit.com/t5/Community/ct-p/EN, accessed on 15 October 2024) was designed to leverage the device’s advanced sensors, such as the gyroscope for motion analysis and the photoplethysmography (PPG) sensor for HR monitoring. Developed using JavaScript (ES14, ECMAScript 2023), the application collects motion data and transmits it in real time to a companion app on a Samsung A10 smartphone. The Fitbit application programming interface (API) and software development kit (SDK) ensure secure and reliable data transfer between the smartwatch and the smartphone, maintaining the integrity of the data. The companion app on the Samsung A10 performs data post-processing using the smartphone’s GPU, offloading computationally intensive tasks from the smartwatch to preserve its battery life. Additionally, an Android application was developed in Java (Java SE 17) to display and store the processed data. This app establishes a local host on the device, allowing fast and secure data transfer between the smartwatch and the smartphone. It also supports data storage and synchronization, enabling users to review their performance history.

### 2.5. Statistical Analysis

Statistical analysis was performed using JMP Pro (Version 17, SAS Institute Inc., Cary, NC, USA) to calculate the ICC for assessing the reliability and agreement between the data collected from the wearable motion sensor, wearable ECG system, and Fitbit. To evaluate the strength of the linear relationships between the Fitbit and the other measurement systems, correlation analyses were conducted, with R^2^- and *p*-values reported. Additionally, *t*-test analysis was conducted to evaluate the absolute differences between measures from the two testing tools. Statistical significance was indicated when *p* < 0.05.

## 3. Results

### 3.1. Participants and Clinical Measures

We collected data from two distinct cohorts for motor and HR assessments. For motor data, we recruited 10 males and 24 females, with an average age of 22 ± 3 years, an average height of 168 ± 9 cm, and an average weight of 64 ± 14 kg. For HR data, we recruited 10 males and 23 females, with an average age of 22 ± 3 years, an average height of 168 ± 10 cm, and an average weight of 64 ± 14 kg.

### 3.2. Motor Component: Wearable Sensor vs. Fitbit

**Figure 2:**
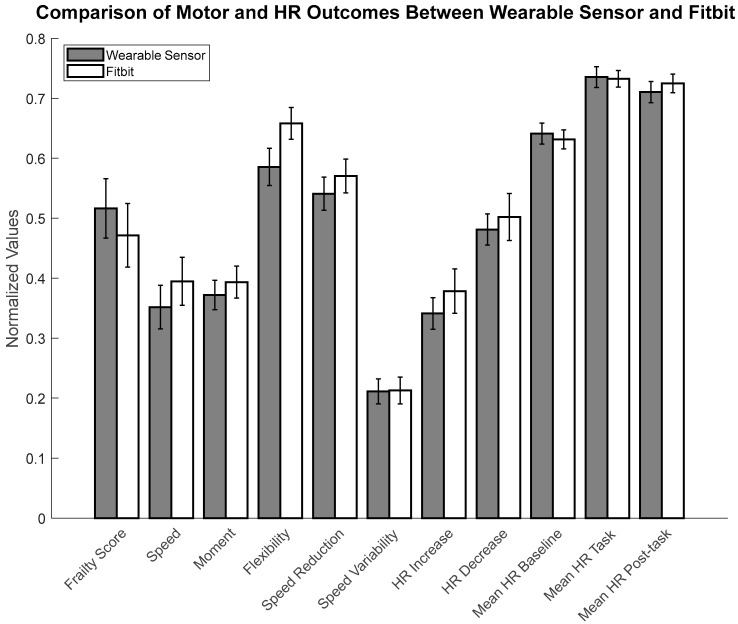
Comparison of motor and HR outcomes between the wearable sensor and Fitbit. All values were normalized to the maximum recorded value for each outcome. Error bars represent the normalized standard error for each outcome.

**Figure 3:**
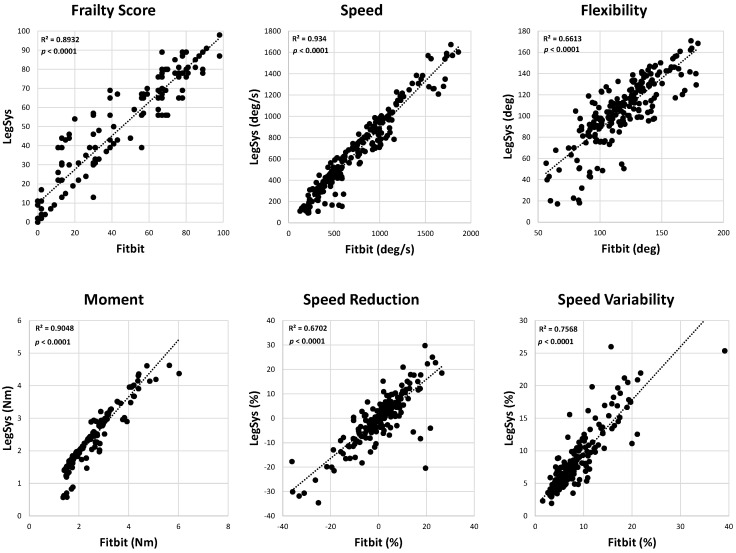
Correlation plots between Fitbit and commercial wearable motion sensor for motor parameters, including frailty score, speed, flexibility, moment, speed reduction, and speed variability. Each subplot includes the *p*-value and R^2^-value for the linear Pearson correlation between the devices.

**Figure 4:**
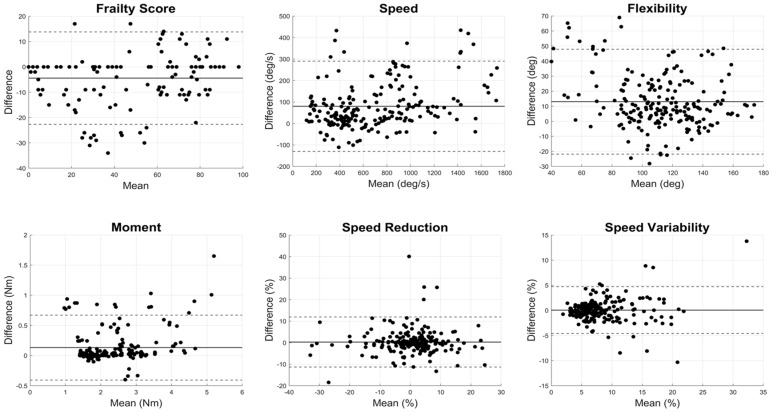
Bland–Altman plots for motor parameters comparing Fitbit and a commercial wearable motion sensor, including frailty score, speed, flexibility, moment, speed reduction, and speed variability. Each subplot displays the mean difference and limits of agreement between the two systems. The *x*-axis represents the mean value of the two measurements (wearable sensor and Fitbit) for each parameter, while the *y*-axis shows the difference between Fitbit and wearable sensor values Solid lines indicate the mean differences, and dashed lines represent the limits of agreement (mean difference ± 1.96 standard deviations).

The results demonstrate excellent agreement for all outcomes related to motor function with ICC values larger than 0.80, except for flexibility (Table 2 and Figure 2). The correlation plots between the Fitbit and the wearable sensor for motor performance outcomes indicated significant linear relationships across all metrics (*p* < 0.0001 and R^2^ = 0.80 ± 0.12; Figure 3). The Bland–Altman plots for motor parameters revealed that the mean differences between the systems were relatively small, and the limits of agreement indicated small variability (Figure 4). The *t*-test *p*-values for the motor parameters showed no significant differences for the frailty score, moment, speed reduction, and speed variability (*p* > 0.1). However, speed (*p* = 0.04) and flexibility (*p* < 0.001) showed differences, suggesting variability between the devices in capturing these parameters (Table 2).

### 3.3. HR Component: ECG vs. Fitbit

**Figure 5:**
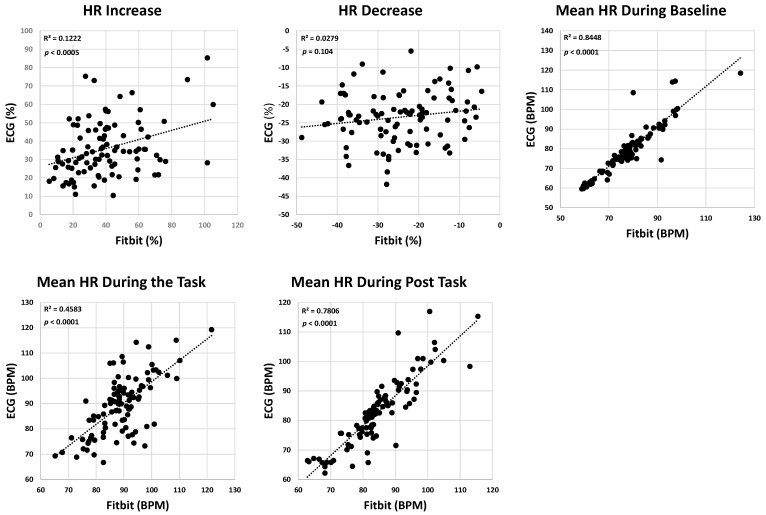
Correlation plots between the ECG system and Fitbit for HR parameters, including HR increase, HR decrease, mean HR during baseline, mean HR during the task, and mean HR during post-task. Each subplot includes the *p*-value and R^2^-value to assess the linear Pearson correlation between the devices.

**Figure 6:**
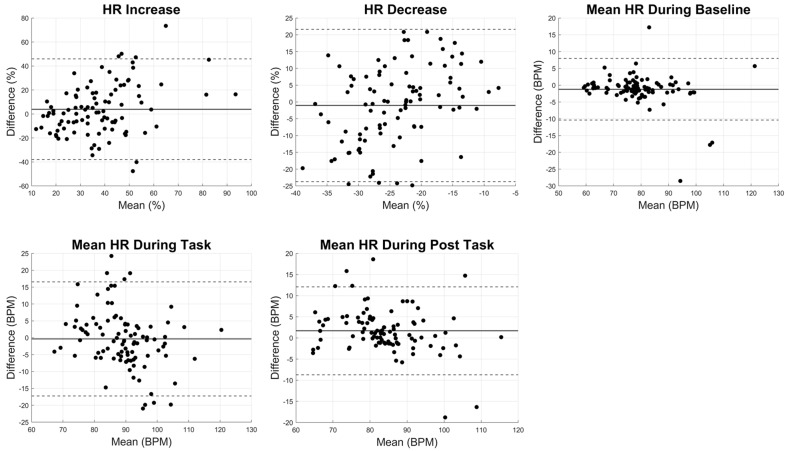
Bland–Altman plots for HR parameters comparing ECG and Fitbit systems, including HR increase, HR decrease, mean HR baseline, mean HR task, and mean HR post-task. Each subplot displays the mean difference and limits of agreement between the two systems. The *x*-axis represents the mean value of the two measurements (ECG and Fitbit) for each parameter, while the *y*-axis shows the difference between Fitbit and ECG system. Solid lines indicate the mean differences, and dashed lines represent the limits of agreement (mean difference ± 1.96 standard deviations).

The ICC values for HR increase and HR decrease were 0.32 and 0.16, respectively, suggesting weak agreement between the wearable ECG sensor and Fitbit recording. However, the agreements for the baseline, task, and post-task mean HR values were moderate to strong, with corresponding ICC values of 0.91, 0.67, and 0.86. The correlations between the Fitbit and the ECG system showed that while the mean HR during baseline, task, and post-task demonstrated moderate to strong correlations (R^2^ = 0.69 ± 0.21), the correlation for HR increase and HR decrease was weak (R^2^ of 0.12 and 0.03, respectively; Figure 5). The *p*-values for the linear correlation were significant for all HR parameters except HR decrease (Figure 5). The Bland–Altman plots for the HR parameters revealed wider limits of agreement, particularly for HR increase and HR decrease, indicating that the Fitbit provided greater variability in capturing the HR dynamics during activity and recovery compared with steady-state conditions (Figure 6). The *p*-values for the *t*-tests for the HR parameters indicated no significant difference between the Fitbit and ECG systems (*p* > 0.1439; Table 2).

## 4. Discussion

As hypothesized, the results demonstrate excellent agreement for the motor parameters, with strong ICC values above 0.8 and significant correlations between Fitbit and the commercial wearable sensor, except for the flexibility measure. One potential reason for the lower agreement in flexibility measurements could be the differing noise levels between the two sensors. When these data are integrated, even slight variations in the signal quality or frequency can become amplified. This effect is particularly relevant for flexibility, as this measurement is more susceptible to external factors; even small inconsistencies in sensor alignment can lead to more noticeable variations in the data due to the integration process for calculating the angular displacement. Consequently, flexibility may show less reliability across devices compared with other motor parameters. Although both devices share a 100 Hz sampling rate, differences in signal-processing algorithms, such as filtering and integration methods, likely contributed to the observed discrepancies. Fitbit may apply proprietary filtering techniques optimized for usability, which can lead to variations in the measured range of motion compared with the commercial sensor.

Additionally, the baseline and post-task HR parameters showed excellent reliability, with ICC values of 0.91 and 0.86, respectively. The weaker agreement for HR increase during activity and HR decrease during recovery, reflected in the lower ICC values, was likely due to limitations in measuring dynamic changes in HR using PPG, a known issue that has been reported in previous research [22,23]. While ECG is the gold standard for HR monitoring, it requires chest electrodes, making it less practical for daily or in-hospital use compared with wrist-worn, PPG-based smartwatches like Fitbit. This trade-off between accuracy and convenience is important to consider. Nevertheless, the frailty score developed from the UEF task relies primarily on motion data, which showed an excellent correlation between sensors. Although the HR data from Fitbit was not as accurate as the motion data, there are two key reasons why using Fitbit HR data still enhanced the frailty score. First, this study demonstrated strong correlations for the HR parameters during the stationary periods of the experiment, suggesting that measures relying on average HR values could be reliable for assessing frailty. Second, while differences between the Fitbit and ECG data were observed, the consistency of these differences across participants may still provide an opportunity to develop a reliable frailty score using Fitbit HR data.

### 4.1. Motor Assessment Using Smartwatch

Our findings demonstrate strong agreement between Fitbit and the commercial wearable sensor for motor performance parameters, confirming the reliability of a smartwatch in tracking physical activity. With ICC values above 0.8 for most motor performance measures, Fitbit proved to be an effective tool for frailty assessment through motion tracking. The *t*-test *p*-values suggested that there were differences in the data collected from Fitbit compared with the commercial gyroscope-based sensor. Specifically, Fitbit tends to capture faster movements and larger ranges of motion (Table 2). These differences likely arise from variations in sensor calibration and data processing between the devices. To account for these variations, further investigations are needed to better understand the sources of discrepancies between Fitbit and commercial sensors, particularly for parameters like flexibility. This will help ensure consistent and accurate comparisons between devices for frailty assessments.

Smartwatches have emerged as a powerful tool for motion tracking, with numerous studies demonstrating their potential to monitor and assess physical activities across various health conditions. These devices are particularly valuable due to their embedded sensors, such as accelerometers and gyroscopes, which enable precise motion detection and classification. For example, one study explored the potential of using a commercial smartwatch to assess upper extremity function in patients with musculoskeletal injuries [24]. The smartwatch was used to capture acceleration and angular velocity data during various upper limb tasks. Metrics related to elbow flexion/extension, forearm supination/pronation, and shoulder rotation demonstrated significant correlations with clinical outcome measures like the upper extremity functional index (UEFI) score. Another study combined a smartwatch with a machine learning model to monitor home-based rehabilitation for chronic stroke survivors [25]. Using accelerometer and gyroscope data, the smartwatch accurately recognized specific rehabilitation exercises, significantly improving patient outcomes. Similarly, a lightweight system was introduced to leverage smartwatch IMU sensors to track wrist motion in real time [26]. Although IMU sensors can experience drift, the system counteracted this by using a smartphone as an acoustic anchor, improving the accuracy of wrist motion tracking. Motion sensors in smartwatches have also been successfully used to detect and classify falls in elderly individuals, achieving a 99.64% accuracy in fall detection through machine learning algorithms [27]. These studies highlight the capability of smartwatches to track motion parameters with high accuracy and reliability.

### 4.2. HR Assessment Using a Smartwatch

Previous research has indicated significant correlations between frailty and HR parameters, such as HRV [8]. HRV is a critical indicator of autonomic nervous system function and overall cardiovascular health, reflecting the balance between sympathetic and parasympathetic activity. However, there is currently no established index that directly associates HR with frailty status. In our previous work, within the UEF and walking testing, we showed a significant association between HRV and HR dynamics with frailty status [3,4,28]. In this study, we aimed to explore the relationship between HR parameters measured by a smartwatch and those recorded by a wearable ECG system to evaluate the feasibility of using smartwatches for future frailty assessment. Our analysis highlights the potential of Fitbit to reliably capture baseline and post-task HR metrics, demonstrating strong agreement with the ECG system. While Fitbit showed limitations in tracking changes in HR, its overall performance underscores its value as a convenient tool for monitoring cardiac autonomic performance in various settings. The *t*-test results support this by showing no significant differences between the two systems for HR increase and decrease despite weaker agreement in the dynamic HR measures. Similarly, for the baseline, task, and post-task HR parameters, there were no significant differences, confirming that Fitbit provides reasonably accurate estimates for steady-state HR measurements.

The current findings are in line with prior studies on PPG-based smartwatches, which have highlighted their limitations during physical activities. For example, one study using the Samsung Gear Sport smartwatch found high accuracy for HR and HRV parameters during sleep but reduced accuracy during physical activity [23]. Similarly, another study compared the Samsung Watch 4’s PPG measurements with those from medical-grade ECG devices and reported deviations in HRV features during physical activities, especially in frequency-domain metrics like pNN50 and low- and high-frequency (LF and HF) analysis [22]. These challenges underscore the limitations of smartwatches in dynamic conditions, as observed in our study. While they perform well in passive or stationary states, their accuracy diminishes during movement, complicating their use for more active monitoring. Notably, in our research, we took measures to reduce motion artifacts during data collection by having participants wear the Fitbit on the arm that was not moving while performing the UEF task. Our results suggest that while there is potential for combining motion and HR data to calculate frailty, further advancements in the dynamic measurement of HR changes and signal quality assessment are needed to improve the reliability of smartwatch-based measurements.

Previous works explained the fundamental differences between ECG and PPG for HRV analysis [29,30]. ECG captures the electrical activity of the heart by detecting R-wave peaks, providing a direct measurement of heartbeat intervals. In contrast, PPG is an optical technique that measures blood volume changes in peripheral tissues, detecting pulse waveforms as blood flows through vessels. This study found that, under resting conditions, pulse rate variability (PRV) derived from PPG correlates closely with HRV from ECG. However, this correlation weakens after exercise, particularly in the high-frequency (HF) range, due to PPG’s sensitivity to factors like respiratory movement and arterial elasticity, which do not affect ECG.

Furthermore, other research underscores the limitations associated with PPG technology, especially in wearable devices designed for continuous health monitoring [31,32]. PPG is particularly vulnerable to inaccuracies caused by various factors, including individual differences such as skin tone, obesity, and age, which can alter light absorption and signal quality. Additionally, physiological variations like respiratory-induced modulation and venous pulsations, as well as external factors such as motion artifacts, ambient light interference, and inconsistent device pressure on the skin, highly impact PPG accuracy. These challenges limit PPG’s reliability for complex cardiovascular measurements beyond basic heart rate monitoring, such as blood pressure estimation and heart rate variability analysis.

### 4.3. Implications for Clinical Practice

The strong agreement between Fitbit and specialized sensor systems for motor parameters suggests that smartwatches could be integrated into clinical practice for frailty assessment. Unlike expensive wearable devices, Fitbits are relatively inexpensive and widely accessible, making them a cost-effective option for both clinical and home settings. Additionally, the use of UEF tests, which assess motor performance through rapid elbow flexions, offers a practical advantage for bed-bound patients as it does not require walking and provides an objective measure of frailty. Given the high ICC values for motor parameters and the reliability of baseline and recovery HR data, Fitbit could be a valuable tool for frailty assessment. This is particularly important for older adults and patients at risk of frailty, as frailty identification may be noticeably simpler using smart devices rather than commercial sensors [4].

### 4.4. Limitations and Future Directions

There were limitations in this study. First, this study was conducted on a healthy, young adult sample, which may limit the generalizability of the findings to older adults or frail individuals, where the variability in HR and motor function may differ. To mimic older adult behaviors, we incorporated external weight into our testing protocol, resulting in a wide range of frailty scores ranging from 0 to 98, based on our previously established models among older adults. Future studies should include older, frail populations to validate the findings in the target demographic. Additionally, while Fitbit performed well in capturing motor parameters, its HR measurements during dynamic tasks showed weaker agreement with the ECG system. Although no significant differences were found between the two systems, Fitbit’s accuracy in tracking HR during dynamic changes requires further improvement. This highlights the need for continued development in wearable HR-monitoring technology, especially during physical activity. Furthermore, this study focused solely on a single smartwatch (i.e., Fitbit Sense 2). Future research could compare various consumer-grade wearables to identify the most suitable device for comprehensive frailty assessments.

## 5. Conclusions

This study established the reliability of Fitbit as a valuable tool for assessing frailty through both motor and HR parameters. For the motor component, excellent agreement was found between Fitbit and the commercial wearable sensor, evidenced by high ICC values exceeding 0.8 across all motor function outcomes, except for flexibility. Strong linear relationships were observed between the Fitbit and commercial wearable sensor, supported by the correlation coefficients (R^2^ = 0.66–0.94) and minimal variability. In terms of HR assessment, Fitbit demonstrated a promising ability to capture baseline, task, and recovery HR, showing strong agreement with the ECG system, with ICC values of 0.91, 0.67, and 0.86, respectively. However, its ability to detect dynamic HR changes during physical activity was lower, as indicated by the weaker ICC values of 0.32 for HR increase and 0.16 for HR decrease. Although the *t*-test results showed no significant differences between the two systems for the HR parameters, this suggests that while Fitbit provides useful HR data for steady-state conditions, the PPG sensor within the watch may not capture rapid fluctuations as precisely as commercial ECG systems. This underscores the need for further refinements in wearable HR-monitoring technologies, especially for dynamic heart rate assessment. Overall, the findings support the reliability of Fitbit for frailty assessment, highlighting its potential as a user-friendly and accessible option for monitoring physical activity and HR. Future studies should explore its effectiveness in older and frail individuals to enhance its generalizability and validate its applicability across diverse health conditions.

## Figures and Tables

**Table 1:** Motor performance and HR parameters definitions [4,20].

Parameter	Definition
Motor Performance Parameters	
Speed (deg/s)	Mean value of the forearm angular velocity range (maximum minus minimum speed) during the 20 s of flexion
Moment (Nm)	Mean value of the maximum moment on the elbow within each flexion/extension, estimated from the moment of inertia of the forearm and the hand
Flexibility (deg)	Mean value of the forearm angular range
Speed reduction (%)	Coefficient of variation (standard deviation divided by the mean) of the angular velocity range
Speed variability (%)	Difference in the angular velocity range between the last and the first 5 s of elbow flexion expressed as a percentage of the initial angular velocity range
HR Parameters	
HR increase (%)	Percentage increase in HR during the task compared with the minimum baseline HR
HR decrease (%)	Percentage decrease in HR during the recovery period compared with the maximum HR during the task
Mean baseline HR (BPM)	Mean HR during the baseline period, which reflects the 60 s of resting state
Mean task HR (BPM)	Mean HR recorded during the UEF task
Mean post-task HR (BPM)	Mean HR during the recovery period, measured for 60 s following the task

**Table 2:** Mean ± standard deviation (SD) values and ICC for motor and HR parameters.

Parameter	Wearable Sensors	Fitbit	ICC	*p*-Value (*t*-Test)
Motor Parameters				
Frailty score (0–100)	50.61 (26.63)	46.20 (28.49)	0.93	0.1087
Speed (deg/s)	655.19 (372.35)	735.22 (408.02)	0.94	0.0401
Moment (Nm)	2.24 (0.81)	2.37 (0.88)	0.94	0.1170
Flexibility (deg)	104.98 (30.47)	118.01 (25.94)	0.71	<0.001
Speed reduction (%)	0.21 (9.77)	0.53 (9.89)	0.82	0.7442
Speed variability (%)	8.26 (4.49)	8.32 (4.77)	0.87	0.8917
HR Parameters				
HR increase (%)	35.87 (15.17)	39.79 (21.31)	0.32	0.1439
HR decrease (%)	−23.45 (6.97)	−24.47 (10.47)	0.16	0.4266
Mean HR baseline (BPM)	79.60 (11.87)	78.40 (10.71)	0.91	0.4606
Mean HR task (BPM)	89.41 (11.55)	89.06 (9.25)	0.67	0.8171
Mean HR post-task (BPM)	83.07 (11.31)	84.76 (9.88)	0.86	0.2730

## Data Availability

The database used for the current study is available upon reasonable request. Please contact the corresponding author, Nima Toosizadeh, at nima.toosizadeh@rutgers.edu for more information.
